# Multiscale Radiometric Stability Analysis of Water Bodies in Multispectral Remote Sensing Imagery

**DOI:** 10.3390/s26051564

**Published:** 2026-03-02

**Authors:** Yanze Yang, Xiankun Ge, Jingjing Chen, Mengjie Xu, Lei Yang

**Affiliations:** School of Instrument Science and Opto-Electronics Engineering, Hefei University of Technology, Hefei 230009, China; 2019213080@mail.hfut.edu.cn (Y.Y.); 2025170077@mail.hfut.edu.cn (X.G.); xumj@hfut.edu.cn (M.X.); yanglei@hfut.edu.cn (L.Y.)

**Keywords:** spatial scale transformation, radiometric stability, rayleigh scattering, flux-conserving resampling

## Abstract

**Highlights:**

**What are the main findings?**

- Spatial resampling prior to data fusion can introduce substantial radiometric distortions in aquatic remote sensing, particularly at coarse spatial resolutions.
- Flux-conserving resampling consistently preserves radiometric stability across scales.

**What are the implications of the main findings?**

- Enforcing flux conservation during resampling is critical for maintaining the integrity of weak aquatic signals and improving the reliability of water quality retrievals.
- The results provide a physically grounded reference for multi-source aquatic data fusion and cross-calibration of sensors with differing spatial resolutions.

**Abstract:**

In remote sensing, multi-sensor data fusion enhances environmental monitoring by integrating complementary observations. A critical step in this integration is spatial resampling to a common scale. Although often regarded as a routine preprocessing operation, resampling can become a significant source of radiometric uncertainty, systematically altering scene radiance during scale transformation, especially in heterogeneous aquatic environments. In this study, we evaluate resampling-induced radiometric uncertainty and assess the physical advantages of flux-conserving resampling in multi-scale aquatic remote sensing. Using the radiometrically stable Landsat 8 OLI sensor as a reference platform, this study develops a radiometric stability–based framework to evaluate multi-scale resampling methods. Radiometric consistency in the visible bands was first evaluated using a Rayleigh scattering calibration, allowing a systematic comparison of four resampling methods across multiple spatial scales. Normalized water-leaving radiance was then retrieved using the Satellite Signal in the Solar Spectrum (6S) radiative transfer model and validated against in situ AERONET-OC measurements. Our results indicate that radiometric consistency decreases with increasing scale, while flux-conserving resampling maintains higher stability and preserves the spatiotemporal characteristics of water radiance. These findings highlight the importance of flux-conserving resampling for multi-scale radiometric fidelity and establish the proposed framework as a reference for reliable multi-source data fusion and quantitative inversion in aquatic remote sensing and beyond.

## 1. Introduction

Satellite remote sensing plays a crucial role in monitoring Earth’s systems [1,2]. Among its applications, multispectral imagery serves as a fundamental tool for quantitative environmental analysis [3]. This capability is particularly evident in ocean color remote sensing, where spectral reflectance supports large-scale and cost-effective monitoring of water quality [4]. However, dedicated ocean color sensors often suffer from coarse spatial resolution, which limits their ability to monitor dynamic processes in coastal and inland waters [5]. Satellites such as Landsat provide relatively higher spatial resolution (30 m), stable calibration, and global coverage. These features make them valuable data sources for aquatic studies [6,7,8]. Nevertheless, passive optical sensors face inherent trade-offs among spatial, spectral, and temporal resolution because photon collection limits and signal-to-noise constraints [9]. Consequently, observations from a single satellite platform are often insufficient for comprehensively characterizing aquatic optical properties, particularly in optically heterogeneous environments and across varying spatial scales.

To overcome these limitations, multi-source data fusion has become an increasingly common strategy to integrate complementary sensors and achieve enhanced spatio-spectral resolution [10]. Extensive research has addressed multi-source data fusion, particularly in relation to spatiotemporal integration [11] and the harmonization of heterogeneous datasets with differing spatial resolutions [12,13]. Among the technical considerations for multi-source fusion, a fundamental requirement is spatial scale conversion, which involves resampling imagery from different sensors onto a common grid [14]. Despite its ubiquitous use, resampling is often treated as a purely technical or geometric operation. In practice, however, it can substantially alter spectral information and compromise pixel-level radiometric integrity, particularly in optically heterogeneous aquatic environments [15]. Previous multi-scale analyses of water bodies have shown that the choice of resampling algorithm affects both the preservation of spatial detail and the consistency of spectral and radiometric signals [16]. Similar effects have also been reported in high-resolution UAV studies, where spatial resolution and resampling strategies were shown to influence classification accuracy and object discrimination [17]. These effects can directly propagate into downstream quantitative products, such as water-leaving radiance, yet they remain insufficiently examined in aquatic remote sensing workflows.

In practice, most studies rely on computationally efficient interpolation-based methods, such as bilinear interpolation, cubic convolution, or Lanczos resampling [18,19]. Although widely adopted, these conventional interpolation methods do not preserve radiative flux during spatial scale transformation and may distort scene-integrated radiance, leading to systematic radiometric and spectral errors. For instance, Tian et al. showed that as spatial resolution decreases, traditional interpolation tends to reduce the depth and area of key spectral features, producing quantifiable spectral distortions compared with more physically informed resampling methods [20]. Importantly, such errors are not limited to traditional image processing workflows: they can also propagate into downstream tasks, such as the quality of synthesized data used in deep learning–based super-resolution training, thereby affecting analytical performance [21]. In contrast, flux-conserving resampling is grounded in the physical principle of energy conservation and preserves total radiance during spatial scale transformation, mitigating these errors. Its effectiveness has been demonstrated in marine remote sensing for harmonizing observations from heterogeneous sensors within virtual constellations [22], as well as in atmospheric remote sensing, where flux-conserving downscaling improves trace gas retrieval accuracy by maintaining radiometric consistency [23].

Despite the promising potential of flux-conserving methods across various remote sensing domains, a systematic evaluation of resampling methods specifically designed for multi-scale aquatic remote sensing remains understudied. In particular, there has been limited effort to evaluate how different resampling methods affect radiometric stability across spatial scales or how such effects propagate into physically meaningful end products, such as normalized water-leaving radiance.

In this study, we focus on the methodological role of spatial resampling as a non-negligible source of radiometric variability in water color remote sensing. Using Landsat-8 OLI data, this study assesses four commonly applied resampling methods, namely bilinear interpolation, cubic convolution, Lanczos interpolation, and flux-conserving resampling, across multiple spatial scales (500 m, 1000 m, and 2000 m). Radiometric stability in the visible bands is first examined using a calibration framework based on Rayleigh scattering. The influence of resampling on normalized water-leaving radiance is then quantified with the 6S radiative transfer model and validated against in situ AERONET-OC observations.

This study demonstrates that spatial resampling is a critical methodological component rather than a simple technical preprocessing step in multi-scale aquatic remote sensing, by directly linking the choice of resampling strategy to radiometric consistency and water color retrieval accuracy. The results highlight the advantages of flux-conserving resampling for maintaining radiometric fidelity across scales and provide a practical framework for implementing reliable, radiometrically consistent multi-source data fusion in aquatic environments.

## 2. Study Area and Data

### 2.1. Overview of the Study Area

**Figure 1:**
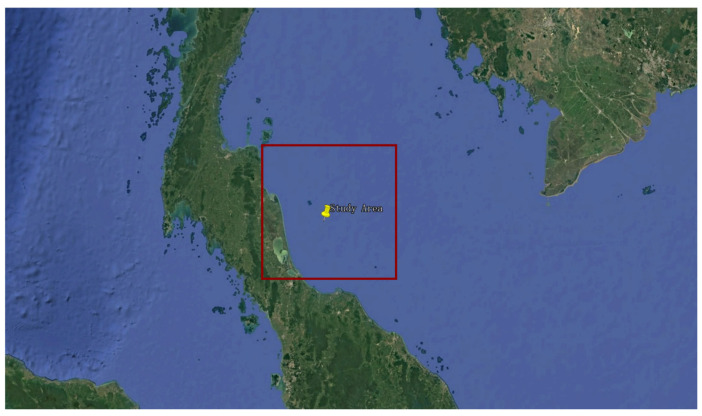
Satellite imagery from Google Earth showing the location of the study site. The study area is delineated by the red box, and its approximate geographic center is indicated by the yellow pushpin.

In this study, the nearshore waters of the eastern Gulf of Thailand (7.5–9.5° N, 100.0–102.0° E; Figure 1) were selected as the primary study area. This region, situated between the Indochina and Malay Peninsulas, comprises a typical tropical semi-enclosed shallow bay with an average depth of 40–60 m and a gentle bathymetric slope, characteristic of continental shelf seas [24]. The strong influence of the Chao Phraya River discharge generates a pronounced optical gradient from highly turbid nearshore waters to clearer offshore waters.

The region is influenced by the Asian monsoon, which drives seasonal variations in hydrographic and atmospheric conditions. Under the northeast monsoon during the dry season (November–February), precipitation and river discharge are reduced, and atmospheric conditions are relatively stable with low aerosol loading, resulting in more homogeneous water optical properties. These relatively stable atmospheric and hydrographic conditions reduce uncertainties in atmospheric correction, thereby providing an ideal natural setting for controlled validation of radiometric calibration and water color remote sensing algorithms. In contrast, the wet season is characterized by higher rainfall and stronger river inflow, which increase water turbidity and variability in atmospheric aerosol conditions. Therefore, the study focused on dry-season conditions to ensure controlled and reliable radiometric validation, thereby minimizing the confounding effects of atmospheric complexity.

### 2.2. Data Sources

The Landsat-8 Operational Land Imager (OLI) scene used in this study was acquired on 15 December 2015 (Path/Row: 128/54). Landsat-8 operates in a sun-synchronous orbit at an altitude of 705 km with a 16-day revisit cycle [25]. The OLI sensor provides multispectral data at 30 m spatial resolution (15 m for the panchromatic band) across a 185 km swath, featuring improved radiometric calibration accuracy and the signal-to-noise ratio compared to previous Landsat missions [26]. Although the scene was acquired in 2015, it was selected because it captured optimal atmospheric and water conditions during the dry season, providing a reliable reference for evaluating the resampling algorithms. The spectral characteristics of the OLI bands are summarized in Table 1, including the coastal aerosol band (443 nm), which is particularly important for aquatic applications.

To evaluate the performance of the resampling methods across different water conditions, three additional AERONET-OC sites were included: MVCO (41.3° N, 70.6° W), Helsinki Lighthouse (59.9° N, 24.9° E), and Lucinda (18.5° S, 146.4° E). Landsat-8 scenes for the auxiliary sites were selected primarily based on cloud cover (<10%) without seasonal restrictions. All scenes, including those from the primary study area in the eastern Gulf of Thailand, were used for water-leaving radiance retrieval to assess the performance of the resampling methods. Scene selection followed strict criteria to ensure data quality, and detailed information for all matched scenes was provided in Table 2.

**Figure 2:**
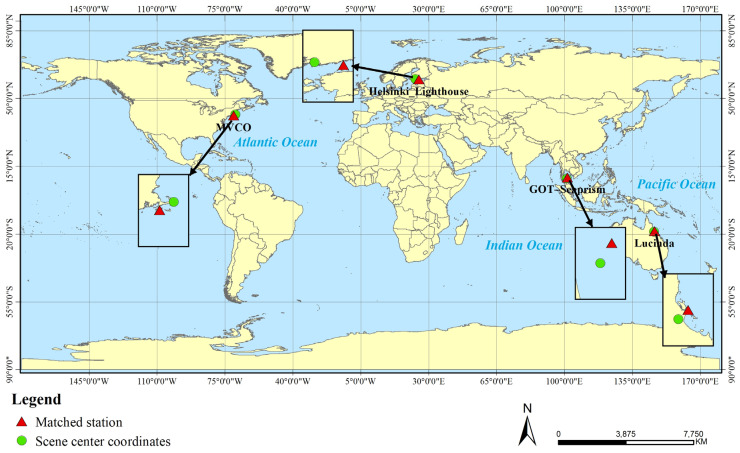
Geographic locations of the four AERONET-OC validation sites and the corresponding centers of the remote sensing images used in this study. The black arrow indicates north, and the black box represents the scale bar.

The primary validation was conducted using the Landsat-8 scene matched to the GOT-Seaprism site (9.3° N, 101.4° E) of the Aerosol Robotic Network for Ocean Color (AERONET-OC) [27], which served as the main reference for radiometric stability analysis. Satellite observations were temporally matched with in situ measurements by averaging all available data acquired within a ±30 min window centered on the satellite overpass time. Data from the three auxiliary sites were used to evaluate the performance of the resampling algorithms across diverse aquatic environments. The geographic distribution of the primary study area and all validation sites is shown in Figure 2.

## 3. Methods

### 3.1. Radiometric Calibration and Atmospheric Correction Models

#### 3.1.1. Radiometric Calibration Model

In this study, Level-1 Landsat-8 OLI data were used as a reference for evaluating radiometric errors introduced by spatial resampling. The OLI instrument has a rigorous radiometric calibration framework, including pre-launch absolute calibration using an integrating sphere and a solar simulator traceable to the National Institute of Standards and Technology (NIST), as well as continuous on-orbit monitoring via a dual solar diffuser system [28]. Such radiometric stability is critical for this study, as it minimizes sensor-induced uncertainties and allows observed radiometric differences to be primarily attributed to spatial resampling effects rather than instrumental artifacts. Top of atmosphere (TOA) radiance was computed from digital number (DN) values using the standard calibration coefficients:

(1) $$\begin{array}{c} L_{\lambda }=\text{gain}*\text{DN}+\text{offset}, \end{array}$$

where $L_{\lambda }$ denotes the TOA radiance, DN represents the digital number of a pixel, and gain and offset are the radiometric calibration coefficients corresponding to the sensor’s gain and bias. The TOA radiance was subsequently converted to TOA reflectance using the following expression:

(2) $$\begin{array}{c} \rho_{TOA}=\frac{\pi {\cdot L}_{\lambda }{\cdot d}^{2}}{{ESUN}_{\lambda }\cdot \cos\left(\theta_{s}\right)}, \end{array}$$

where π converts the directional radiance $L_{\lambda }$ to hemispherical reflectance, $d^{2}$ is the Earth-Sun distance correction factor for orbital eccentricity, ${ESUN}_{\lambda }$ is the mean exoatmospheric solar irradiance, and $\cos\left(\theta_{s}\right)$ corrects for the projection of irradiance onto a horizontal surface based on the solar zenith angle $\theta_{s}$.

#### 3.1.2. Atmospheric Correction Model

A controlled experiment to isolate spatial resampling effects was conducted within the 6S radiative transfer framework using its deterministic ocean surface model under fixed atmospheric conditions. The ocean surface reflectance $\rho_{os}$ was calculated using the standard 6S formulation [29]:

(3) $$\begin{array}{c} \begin{array}{c} \rho_{os}\left(\theta_{s},\theta_{s},\phi ,\lambda \right)=\rho_{wc}\left(\lambda \right)+\left\{1-W\right\}\cdot \rho_{gl}\left(\theta_{s},\theta_{s},\phi ,\lambda \right)+\left\{1-\rho_{wc}\left(\lambda \right)\right\}\cdot \rho_{w}\left(\theta_{s},\theta_{s},\phi ,\lambda \right), \end{array} \end{array}$$

where $\phi_{s}$, $\phi_{v}$ are the solar and sensor azimuth angles, and ${\phi =\phi }_{s}-\phi_{v}$ is the relative azimuth angle. $\rho_{wc}$ is whitecap reflectance, $\rho_{gl}$ is sun glint, $\rho_{w}$ is water-leaving reflectance, and W is whitecap coverage. The ocean surface reflectance was treated as a fixed input, while top of atmosphere reflectance was simulated from this surface model using the 6S radiative transfer calculations, ensuring that variations across spatial resolutions were attributable to pixel aggregation.

#### 3.1.3. Lookup Table Construction

The lookup table (LUT) was constructed using the 6S radiative transfer model to support calibration validation. By precomputing reflectance values across a range of atmospheric and geometric conditions, the LUT holds these factors constant, ensuring that observed radiometric differences between images at different resolutions primarily reflect the effects of spatial resampling.

**Figure 3:**
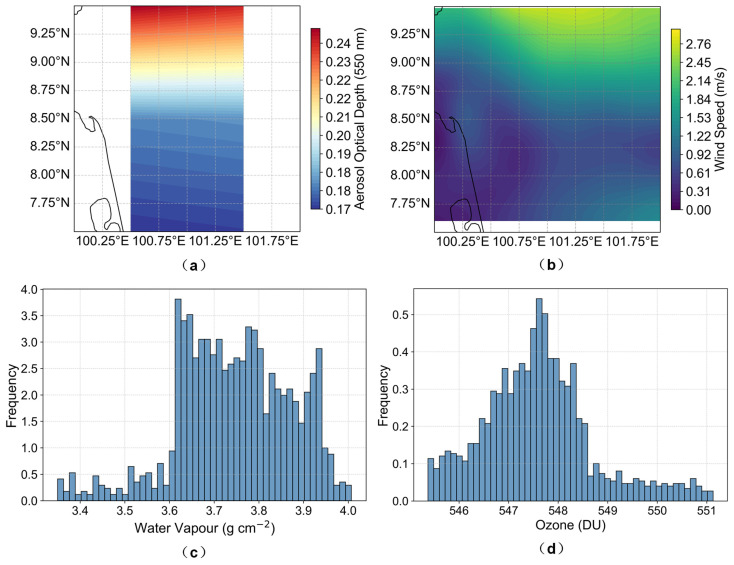
Spatial and statistical distributions of atmospheric parameters over the study area on 15 December 2015: (**a**) AOD, (**b**) wind speed, (**c**) water vapor frequency distribution, and (**d**) ozone frequency distribution. The curves in (**a**,**b**) indicate background geographic features.

For the GOT-Seaprism site, the LUT simulated water-leaving reflectance across the full range of expected conditions, including variations in solar and sensor zenith angles, relative azimuth angles, aerosol loading, and sea surface roughness (represented by wind speed). The LUT was populated with realistic data, including Aerosol Optical Depth (AOD) from MODIS and wind speed, water vapor, and ozone from the ERA5 reanalysis. The ranges of key variables such as AOD and wind speed were determined from statistical analysis over the study area. The chosen discretization steps were set to balance computational efficiency and interpolation accuracy, with step sizes sufficiently fine to capture typical atmospheric and geometric variability. This choice ensures that interpolation-induced errors remain limited, as further quantified in Section 5.1.2. Water vapor (3.75 g · cm^−2^) and ozone (547.46 DU) were set to representative, stable values. In this study, these two atmospheric constituents were held constant to ensure consistency across resampling experiments and to isolate the effect of spatial processing. Although both water vapor and ozone may exhibit spatial variability at larger spatial scales, their horizontal gradients within the selected marine study area are relatively limited under the atmospheric conditions considered. Treating them as spatially uniform, therefore, enables a controlled comparison of radiometric differences induced by resampling, without introducing additional variability from atmospheric heterogeneity. Reflectance for a specific scene was then obtained via linear interpolation within the fixed table. The distributions of LUT input parameters are summarized in Figure 3 and Table 3.

### 3.2. Multiscale Resampling Algorithm

Spatial resampling is a critical step in spatial scale transformation in remote sensing imagery. Ocean color signals are particularly vulnerable to distortions during this process due to their weak water-leaving radiance and high sensitivity to atmospheric and sub-pixel effects. Conventional interpolation methods, which do not conserve radiometric flux, can further exacerbate these distortions, leading to signal attenuation.

In this study, the resampling method was treated as the only controlled variable to quantitatively evaluate its impact on radiometric consistency and ocean color retrieval accuracy. To isolate resampling-induced effects, observation geometry, including BRDF and solar–viewing parameters, atmospheric correction procedures, and masking strategies were kept identical across all experiments, while only the pixel resampling algorithm was varied. Under this controlled framework, a flux-conserving (FC) resampling method was applied, while bilinear, cubic convolution, and Lanczos interpolation were used for comparison in multi-scale experiments.

#### 3.2.1. Flux-Conserving Resampling

Flux-conserving resampling is an image resampling method based on the Sutherland–Hodgman polygon clipping algorithm [30]. It is grounded in the flux-conserving scheme originally proposed by McGlynn [31]. Unlike conventional resampling techniques, it incorporates the physical principle of total flux conservation, ensuring that the total radiometric energy within the observation area remains unchanged during pixel-scale transformation.

In remote sensing, total flux refers to the sum of the radiance values of each pixel multiplied by its corresponding spatial area, representing the total radiometric energy contribution of the entire region. The flux-conserving approach calculates the overlap area between source and target pixels and applies area-weighted accumulation of the original radiance values to the target grid cells. This process preserves radiometric flux and ensures physical consistency during scale conversion.

**Figure 4:**
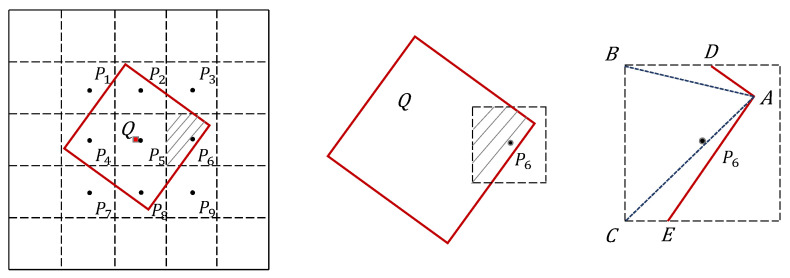
Principle of flux-conserving resampling. The overlap between source pixels $P_{i}$ and target pixel Q is computed using polygon clipping, and contributions are accumulated in an area-weighted manner.

As illustrated in Figure 4, the source pixel grid serves as the clipping window. Each resampled target pixel Q is assumed to overlap spatially with its nine surrounding source pixels ($P_{i},i=1,...,9$). Each overlapping region is decomposed into triangles using a concave polygon clipping algorithm, and the total area of the overlap is calculated. The overlap fraction $S_{i}$ for each source pixel $P_{i}$ relative to Q is then determined. Based on these overlap coefficients, the flux contribution $P^{\prime }\left(i\right)$ of each source pixel to Q is computed, yielding the resampled pixel value $Q^{\prime }$ as the weighted sum of all contributions:

(4) $$\begin{array}{c} S_{i}=\frac{Area\left(P_{i}\cap Q\right)}{Area\left(Q\right)}, \end{array}$$

(5) $$\begin{array}{c} P^{\prime }\left(i\right)=\frac{P_{i}\times S_{i}}{\sum S_{i}}, \end{array}$$

(6) $$\begin{array}{c} Q^{\prime }=\sum_{n}^{i=1,2,...,n}P^{\prime }\left(i\right). \end{array}$$

The flux-conserving algorithm was implemented in Python 3.9 for this study, adapting the core area-weighting scheme. Our implementation supports multi-band imagery and applies proportional clipping to edge pixels, such that only the valid overlapping portions of input pixels contribute to output pixels at boundaries, ensuring radiometric consistency across spatial scale transformations.

#### 3.2.2. Alternative Resampling Methods

1. Bilinear interpolation

**Figure 5:**
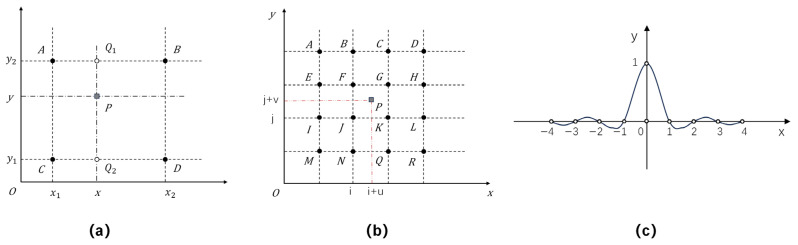
Schematic of three resampling methods: (**a**) bilinear interpolation, (**b**) cubic convolution interpolation, and (**c**) Lanczos kernel (a = 4).

In bilinear interpolation, the value of a target pixel is estimated from the four nearest source pixels (A, B, C, and D) through distance-weighted averaging. As shown in Figure 5a, linear interpolation is first carried out along the *x*-axis between adjacent pixels, followed by interpolation along the *y*-axis, yielding the final value at point P (x, y).

2. Cubic convolution interpolation

Cubic convolution interpolation calculates the value at point P (i + u, j + v) using the 16 pixels within a 4 × 4 neighborhood. Each contribution is weighted according to a cubic convolution function, as illustrated in Figure 5b.

3. Lanczos interpolation

Lanczos interpolation estimates the target pixel value by weighted averaging within a 2a × 2a neighborhood, where a = 4 in this study. As shown in Figure 5c, the weighting is determined by the Lanczos kernel function:

(7) $$\begin{array}{c} L\left(x\right)=\left\{\begin{array}{c} 1,\;x=0\\ \frac{a\sin\left(\pi x\right)\sin\left(\frac{\pi x}{a}\right)}{\pi^{2}x^{2}},\;0<\left|x\right|<a\\ 0,\;otherwise \end{array}\right.. \end{array}$$

The kernel function features a central lobe of width a, flanked by $\left(a-1\right)$ alternating negative and positive side lobes. $I\left(i,j\right)$ denotes the original pixel values. The two-dimensional interpolation for a target pixel is computed as the product of two one-dimensional Lanczos kernels:

(8) $$\begin{array}{c} S\left(x,y\right)=\sum_{i=\left[x\right]-a+1}^{\left[x\right]+a}\sum_{j=\left[y\right]}^{\left[y\right]+a}S_{ij}L\left(x-i\right)\cdot L\left(y-j\right). \end{array}$$

### 3.3. Water Pixel Selection Method

To evaluate the impact of spatial resampling on radiometric stability, we developed a workflow for selecting clean water pixels. Throughout the process, steps such as land masking, cloud detection, and glint filtering ensured stable atmospheric and surface water conditions, so that variations in water pixel radiance could be attributed primarily to the resampling method applied. Finally, water regions were extracted using a dual-band thresholding approach based on the green and near-infrared (NIR) bands.

#### 3.3.1. Land and Cloud Mask

Land and cloud pixels were removed prior to water pixel selection to ensure radiometrically homogeneous conditions. Owing to their distinct spectral characteristics, water pixels typically exhibit low values of the normalized difference vegetation index (NDVI), in contrast to the higher values associated with vegetation and bare soil. Therefore, a land mask was created by applying a threshold of NDVI > 0.08, which was empirically determined through iterative testing to ensure stable land–water separation under local conditions:

(9) $$\begin{array}{c} NDVI=\frac{NIR-RED}{NIR+RED}. \end{array}$$

Here, NIR and RED represent the reflectance in the near-infrared and red bands. Optimal NDVI thresholds may vary in other regions and conditions and should be adjusted accordingly [32].

Cloud pixels are characterized by high reflectance in the SWIR1 band (B6) [33]. Based on analysis of the imagery characteristics in the study area and multiple tests, an empirical threshold was adopted for initial cloud detection:

(10) $$\begin{array}{c} \text{SWIR1}>0.12. \end{array}$$

Under the conditions considered in this study, this threshold reliably identifies cloud pixels while minimizing misclassification of water areas. A morphological closing operation (dilation followed by erosion) was subsequently applied to refine cloud boundaries and generate a spatially coherent cloud mask.

#### 3.3.2. Sun-Glint Filtering

To mitigate specular reflection effects, pixels affected by sun glint were excluded using a geometry-based criterion. The glint angle was computed from solar and sensor zenith and azimuth angles provided in the Landsat-8 metadata. Pixels with glint angles ≤ 40° were removed, following established thresholds reported in previous studies [34].

(11) $$\begin{array}{c} \theta_{g}=\cos^{-1}\left[\cos\theta_{s}\cos\theta_{v}+\sin\theta_{s}\sin\theta_{v}\cos\left(\phi \right)\right], \end{array}$$

where $\theta_{g}$ is the glint angle, $\theta_{s}$ and $\theta_{v}$ are the solar and sensor zenith angles, and ϕ (in Equation (3)) denotes the relative azimuth angle.

#### 3.3.3. Dual-Band Water Pixel Identification

After land, cloud, and glint removal, clear water pixels were identified using a dual-band thresholding approach applied to TOA reflectance. Pixels were classified as water only when reflectance in both the green and NIR bands satisfied predefined thresholds ($\rho_{Green}$ < 0.08 and $\rho_{NIR}$ < 0.15) [35,36], which ensures suppression of atmospheric path radiance and dark non-water targets.

(12) $$\begin{array}{c} \text{WaterPixel}=\left\{\begin{array}{c} 1\;,if\;\rho_{Green}<T_{1}\;and\;\rho_{NIR}<T_{2}\\ 0\;,\;\;otherwise \end{array}\right.. \end{array}$$

Sensitivity tests indicated that moderate variations in threshold values did not affect the relative performance ranking of resampling methods.

### 3.4. Radiometric Stability Validation Methodology

#### 3.4.1. Rayleigh Scattering Calibration

1. Calibration Site Selection and Water Pixel Stability Analysis

To provide a spatially homogeneous and spectrally consistent baseline for radiometric validation, candidate open-water calibration regions were identified using a sliding-window approach designed to minimize land, cloud, and adjacency contamination, ensuring that subsequent radiometric comparisons primarily reflect resampling effects. The analysis was conducted in the UTM projection to maintain metric consistency, with a window size of 100 km × 100 km and a 20 km step to provide spatially representative, overlapping samples. Water pixels within each window were identified using the water mask and preprocessing scheme described in Section 3.3.

The selection procedure involved three steps:

(1) Water coverage screening: to ensure high spatial homogeneity and minimize contamination from land, clouds, or adjacency effects, only windows with water coverage exceeding 90% were retained;
(2) Spatial connectivity validation: an 8-neighborhood connected component analysis was applied, and windows were kept only if the largest contiguous water body accounted for >90% of the total area;
(3) Coordinate conversion: centroids of the selected windows were transformed from UTM to WGS84 geographic coordinates for geolocation.

**Figure 6:**
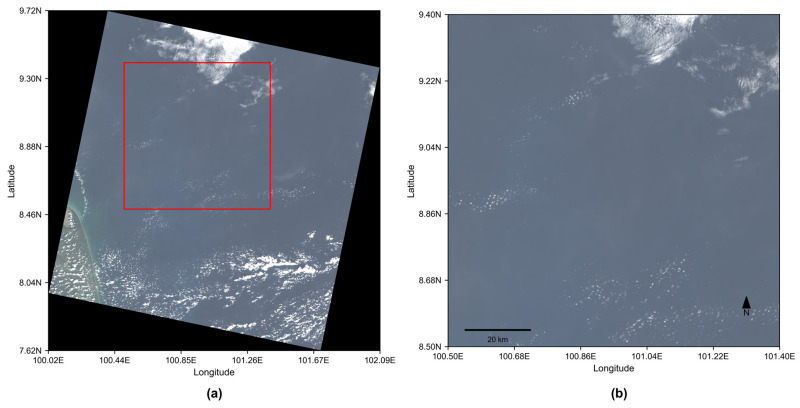
Calibration site selection results based on Landsat-8 RGB composites. (**a**) Spatial distribution of 100 km × 100 km candidate windows meeting the selection criteria. (**b**) Enlarged RGB view of the retained calibration region.

The screening procedure initially yielded 12 candidate windows, from which 4 spectrally homogeneous regions were identified. The window with the highest water coverage among these four was selected as a representative calibration site (Figure 6), with a geographic centroid at 8.95° N, 100.95° E, covering a 100 km × 100 km area and a water coverage of 94.80% at the native 30 m resolution. As shown in Figure 6a, the red box marks the selected calibration site; the black areas indicate regions with no data, and the colored area represents the actual Landsat-8 scene extent.

**Figure 7:**
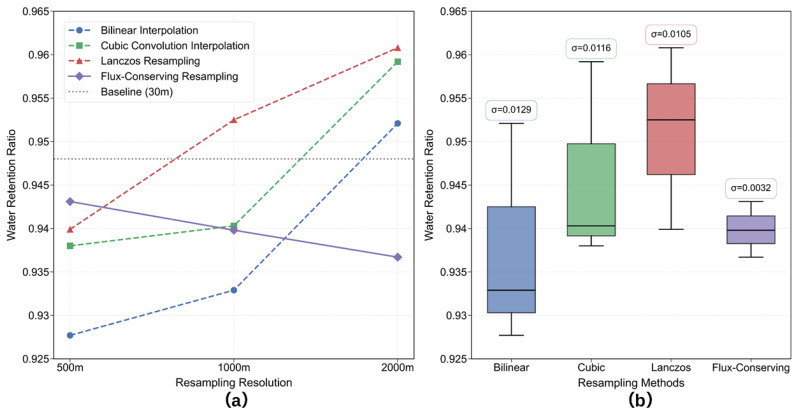
Performance evaluation of resampling methods for water pixel preservation across scales. (**a**) Water pixel preservation rates across spatial scales, (**b**) performance variability assessed through standard deviation (σ).

At the study site, we quantitatively evaluated four spatial resampling methods—bilinear interpolation, cubic convolution, Lanczos, and flux-conserving—for their effects on water pixel spatial consistency at 500, 1000, and 2000 m. Figure 7a reports the proportion of valid water pixels at each resampled resolution relative to the 30 m baseline (0.9480). Figure 7b shows boxplots of water pixel stability; the standard deviation (σ) quantifies variability across scales.

Results show that cubic convolution and Lanczos resampling produce higher proportions of water pixels at coarser resolutions (maximum: 0.9608), primarily due to smoothing of high-frequency textures and boundary blurring. Bilinear interpolation exhibits pronounced instability across scales (σ = 0.0105), indicating limited spatial consistency. In contrast, flux-conserving resampling achieves the highest stability (σ = 0.0026), effectively preserving water pixel structures during scaling. These results demonstrate its superiority for applications sensitive to radiometry.

2. Rayleigh Scattering Radiometric Validation

This approach leverages molecular Rayleigh scattering over the open ocean as an absolute radiometric reference [37]. Validation is performed by comparing Landsat-8 OLI visible-band measurements (483–655 nm) with 6S-simulated values interpolated from precomputed LUTs. The Rayleigh calibration coefficient (A) is defined as follows [38]:

(13) $$\begin{array}{c} A=\frac{\rho_{TOA}^{meas}}{\rho_{TOA}^{cal}}. \end{array}$$

Here, $\rho_{TOA}^{meas}$ represents the observed TOA reflectance from Landsat-8 OLI, and $\rho_{TOA}^{cal}$ denotes the corresponding 6S-simulated TOA reflectance under identical geometric and atmospheric conditions.

**Figure 8:**
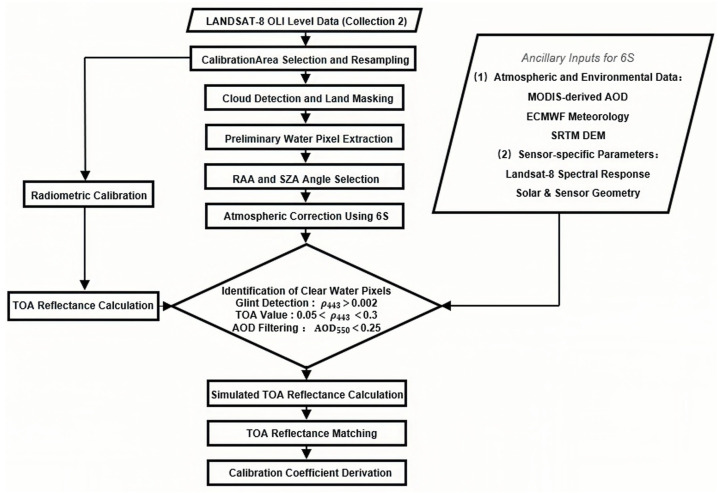
Schematic workflow for deriving the Rayleigh scattering calibration coefficient illustrating the processing chain from Landsat-8 OLI data input to final calibrated outputs.

Based on the models and methods described in the preceding sections in Section 3, the complete workflow for calculating this coefficient is illustrated in Figure 8.

#### 3.4.2. Retrieval of Normalized Water-Leaving Radiance

In ocean color remote sensing, the water-leaving radiance ($L_{w}$) is derived by subtracting surface-reflected radiance and atmospheric scattering contributions from the total radiance measured at the top of the atmosphere [39]. This process is expressed as:

(14) $$\begin{array}{c} L_{w}=\frac{L_{TOA}-t{\cdot L}_{gl}-L_{r}-L_{a}}{t_{d}}-L_{wc}. \end{array}$$

$L_{TOA}$ is the total radiance at the top of the atmosphere; $t_{d}$ and t are the diffuse and direct atmospheric transmittances; $L_{wc}$ and $L_{gl}$ denote the whitecap and glint radiances; $L_{r}$ accounts for Rayleigh scattering and its interactions with aerosols; and $L_{a}$ represents the path radiance due to aerosols.

Atmospheric correction was performed using the 6S radiative transfer model. The procedure involved sequentially removing the contributions of Rayleigh scattering and aerosol path radiance from the top of atmosphere radiance to obtain the sea surface radiance. Subsequently, surface-related contributions, including sun glint and whitecap radiance, were subtracted from the sea surface radiance to obtain the water-leaving radiance at each wavelength.

**Figure 9:**
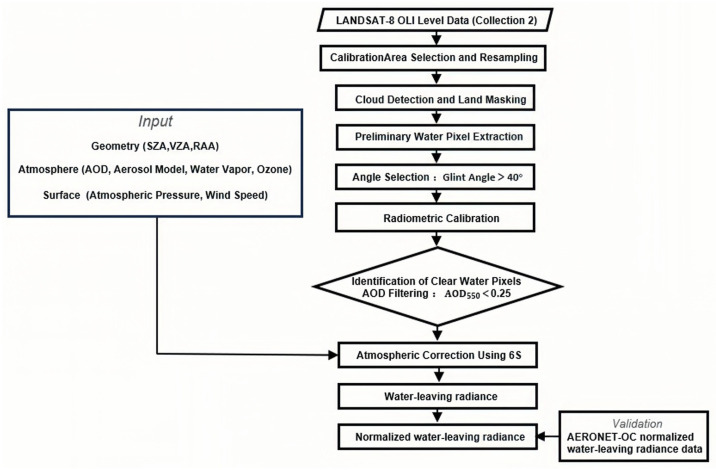
Workflow for retrieving normalized water-leaving radiance from Landsat-8 OLI, including cloud-free water screening, 6S-based radiometric calibration and atmospheric correction, and validation with AERONET-OC data.

To facilitate quantitative comparison of aquatic optical properties across different observation scenarios, the water-leaving radiance at each wavelength is converted to normalized water-leaving radiance. The complete retrieval workflow is illustrated in Figure 9, and the normalized water-leaving radiance is computed according to Wang et al. [40] as:

(15) $$\begin{array}{c} L_{wN}\left(\lambda \right)=L_{w}\left(\lambda \right)F_{0}\left(\lambda \right)/E_{d}\left(0^{+}\right)=\frac{d^{2}L_{w}\left(\lambda \right)}{t{\left(\lambda \right)}^{↓}\left(1-\rho \left(\theta_{s}\right)\right)\cos\left(\theta_{s}\right)}. \end{array}$$

In this equation, $L_{wN}\left(\lambda \right)$ denotes the normalized water-leaving radiance; $F_{0}\left(\lambda \right)$ is the mean extraterrestrial solar irradiance; $E_{d}\left(0^{+}\right)$ represents the downwelling irradiance just above the water surface; $d^{2}$ is the Earth–Sun distance correction factor; $\rho \left(\theta_{s}\right)$ corresponds to the Fresnel reflectance of the sea surface; $t{\left(\lambda \right)}^{↓}$ is the downward atmospheric transmittance; and $\cos\left(\theta_{s}\right)$ accounts for the solar zenith angle.

## 4. Results

### 4.1. Multi-Scale Validation of Rayleigh Scattering Radiometric Stability

The visible bands of Landsat-8 OLI (443 nm, 486 nm, 561 nm, and 655 nm) cover the shortwave spectrum spanning blue to red, where Rayleigh scattering is pronounced. These bands are therefore suitable for evaluating the accuracy of Rayleigh scattering models and the stability of radiometric calibration coefficients. Clean water pixels at 500 m, 1000 m, and 2000 m spatial resolutions were extracted as described in Section 3.3. Together with satellite observation geometry and 6S radiative transfer model LUTs, pixel-level top of atmosphere (TOA) apparent reflectance was simulated.

**Figure 10:**
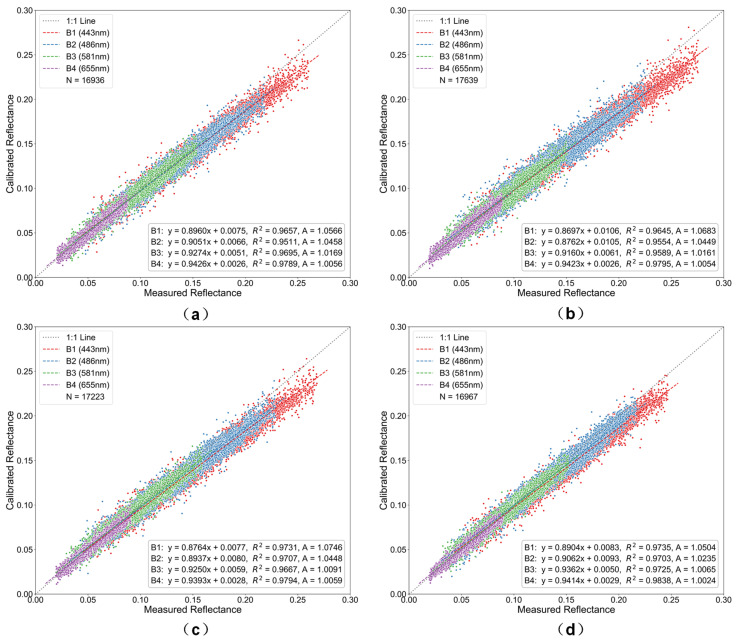
Scatter plots of radiometric calibration results at 500 m resolution for the four visible bands using different resampling methods: (**a**) bilinear, (**b**) cubic convolution, (**c**) Lanczos, and (**d**) flux-conserving. Colors denote the four spectral bands (blue for Band 1, green for Band 2, red for Band 3, and purple for Band 4).

**Figure 11:**
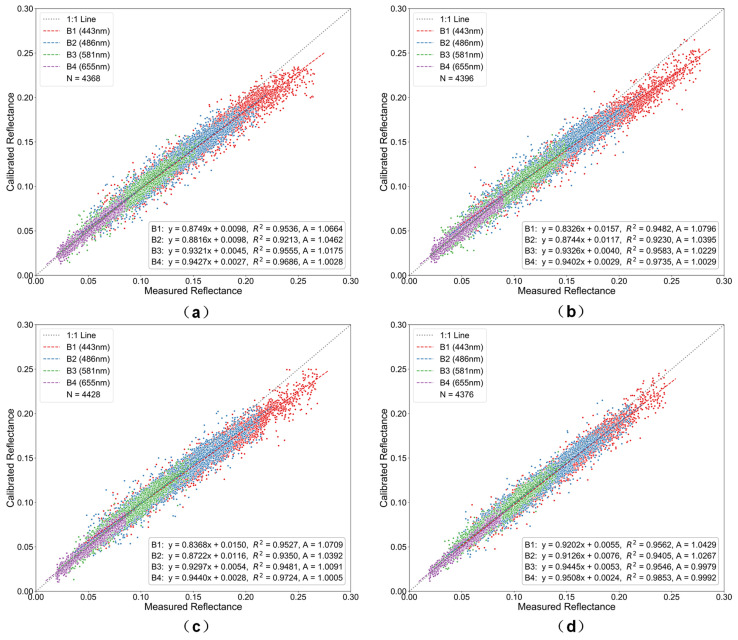
Scatter plots of radiometric calibration results at 1000 m resolution for the four visible bands using different resampling methods: (**a**) bilinear, (**b**) cubic convolution, (**c**) Lanczos, and (**d**) flux-conserving. Colors denote the four spectral bands (blue for Band 1, green for Band 2, red for Band 3, and purple for Band 4).

**Figure 12:**
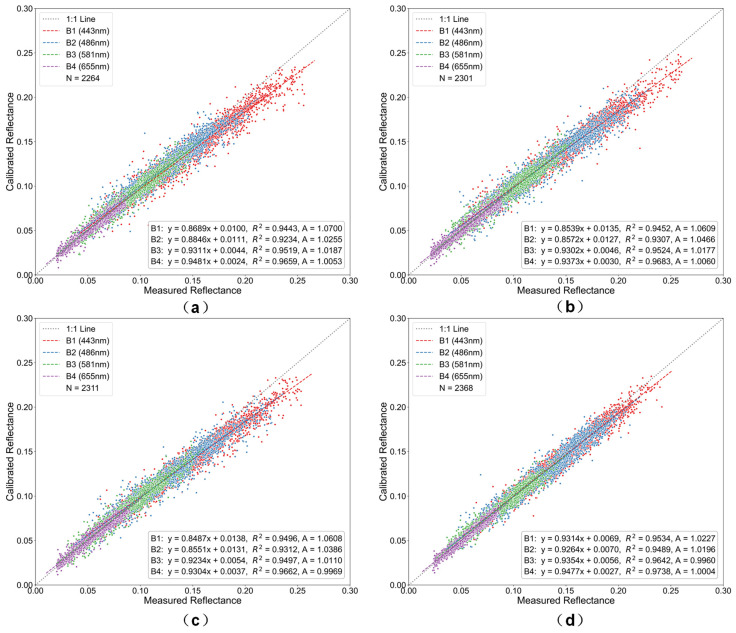
Scatter plots of radiometric calibration results at 2000 m resolution for the four visible bands using different resampling methods: (**a**) bilinear, (**b**) cubic convolution, (**c**) Lanczos, and (**d**) flux-conserving. Colors denote the four spectral bands (blue for Band 1, green for Band 2, red for Band 3, and purple for Band 4).

The Rayleigh scattering calibration coefficient A was derived from the ratio of measured TOA reflectance (based on Landsat-8 OLI radiometric calibration) to 6S-simulated calibrated TOA reflectance (Equation (13)). A linear regression model was then established between these two datasets, as shown in Figure 10, Figure 11 and Figure 12. In the figures, the *x*-axis represents measured TOA reflectance from Landsat-8 OLI, and the *y*-axis represents the 6S-simulated calibrated TOA reflectance. The scatter points correspond to pixel-level data. The solid line represents the fitted regression line, while the dashed line denotes the 1:1 reference line.

Radiometric calibration performance exhibits marked variations among different resampling methods and spatial resolutions (Figure 10, Figure 11 and Figure 12). At 500 m resolution, all methods closely follow the 1:1 reference line, with flux-conserving (FC) resampling showing the tightest clustering. As pixel size increases to 1000 m, observable pixel-mixing effects reduce calibration accuracy, while coarsening to 2000 m leads to pronounced radiometric smoothing, larger deviations, and increased scatter. Method-dependent differences become more evident at coarser resolutions, particularly in the blue bands, where FC resampling outperforms bilinear interpolation by maintaining superior agreement with reference data. Calibration coefficients (A) display wavelength-dependent variations, with larger changes in shorter bands (B1) and minimal variation in longer bands (B4), reflecting the differential sensitivity of spectral bands to resampling.

These observations are consistent with the underlying principles of each resampling technique. The energy-conserving approach of FC resampling mitigates nonlinear error accumulation during scale transformations, whereas bilinear and cubic convolution are more susceptible to subpixel heterogeneity. Lanczos interpolation, while reducing high-frequency artifacts via its sinc-based kernel, does not fully conserve radiometric energy, leading to moderate performance degradation at coarser resolutions.

Overall, FC resampling consistently outperforms alternative methods, preserving radiometric fidelity and maintaining stable calibration coefficients across all spectral bands, even under coarse-resolution conditions with pronounced mixed-pixel effects.

To quantify these observations, relative errors (δ) were calculated to assess radiometric consistency across spatial resolutions for each spectral band as defined in Equation (16):

(16) $$\begin{array}{c} \delta =\left|\frac{\rho_{TOA}^{meas}-\rho_{TOA}^{cal}}{\rho_{TOA}^{meas}}\right|\times 100\%, \end{array}$$

where $\rho_{TOA}^{meas}$ denotes the measured TOA reflectance from Landsat-8 OLI and $\rho_{TOA}^{cal}$ represents the 6S-calibrated TOA reflectance, respectively, consistent with the notation in Equation (13) for the calibration coefficient A.

**Figure 13:**
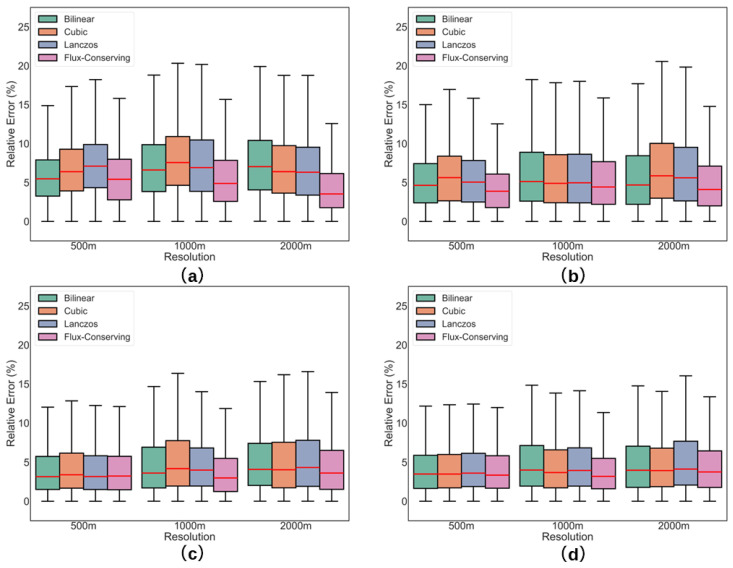
Relative errors of different resampling methods at spatial resolutions of 500 m, 1000 m, and 2000 m across the investigated spectral bands: (**a**) 443 nm, (**b**) 486 nm, (**c**) 561 nm, and (**d**) 655 nm.

For each resampling method at the three investigated spatial resolutions, relative errors were computed for all spectral bands. The results are presented in Figure 13 and Table 4. In Figure 13, the boxplots summarize the statistical distribution of errors: the boxes indicate the interquartile range (IQR, 25th–75th percentiles), the whiskers extend to 1.5 × IQR, the horizontal bars represent the minimum and maximum values within this range, and the solid line within each box indicates the median.

As shown in Figure 13 and Table 4, the flux-conserving (FC) resampling method consistently demonstrated superior performance across all investigated spatial resolutions (500–2000 m) and spectral bands (443–655 nm), achieving both the lowest relative errors and the highest stability. For instance, in the 443 nm band, FC maintained an average relative error of 5.71% at 500 m resolution, increasing only moderately to 6.93% at 2000 m. In contrast, conventional interpolation methods exhibited more pronounced error growth across the same resolution range: bilinear (BL) increased from 5.90% to 7.80%, while cubic convolution (CC) rose from 6.74% to 7.17%. Lanczos interpolation showed performance comparable to FC at finer resolutions (e.g., 5.60% at 500 m for 486 nm), but degraded more noticeably at coarser resolutions.

In addition to its overall accuracy, distinct spectral dependencies were observed in resampling accuracy. Shorter wavelength bands (443 nm and 486 nm) consistently exhibited higher mean relative errors (5.26–7.80%) and greater variability (standard deviations of 3.48–5.31%) compared to longer wavelengths (561 nm and 655 nm), likely due to stronger spatial heterogeneity and increased sensitivity to atmospheric scattering. Notably, FC maintained exceptional consistency across the spectrum, with standard deviations remaining below 4.64% even at the coarsest resolution (2000 m). This observed superiority in radiometric stability aligns with the findings of Lin et al. [41] in multi-sensor data fusion, underscoring the generalizability of the flux-conserving principle for remote sensing applications requiring high radiometric fidelity.

### 4.2. Multi-Scale Water-Leaving Radiance Retrieval Validation

To evaluate the retrieval accuracy and radiometric consistency of the multi-scale methodology, the normalized water-leaving radiance $\left(L_{wn}\right)$ was retrieved and validated at four geographically diverse AERONET-OC sites: GOT-Seaprism, MVCO, Helsinki Lighthouse, and Lucinda. Landsat-8 imagery at 30 m resolution was used as the baseline and resampled to coarser spatial resolutions of 500 m, 1000 m, and 2000 m using four different resampling methods. For each scale, clear-water pixels were identified and atmospherically corrected using the 6S radiative transfer model to obtain $L_{wn}$ in the visible bands. For validation, the mean retrieved $L_{wn}$ values at each scale were calculated to represent the regional radiometric characteristics. These values were then compared with concurrent or nearest-available Level 1.5 measurements from the corresponding AERONET-OC sites, using the temporal matching window defined in Section 2.2. This procedure enables assessment of both scale-dependent retrieval performance and absolute radiometric accuracy.

**Figure 14:**
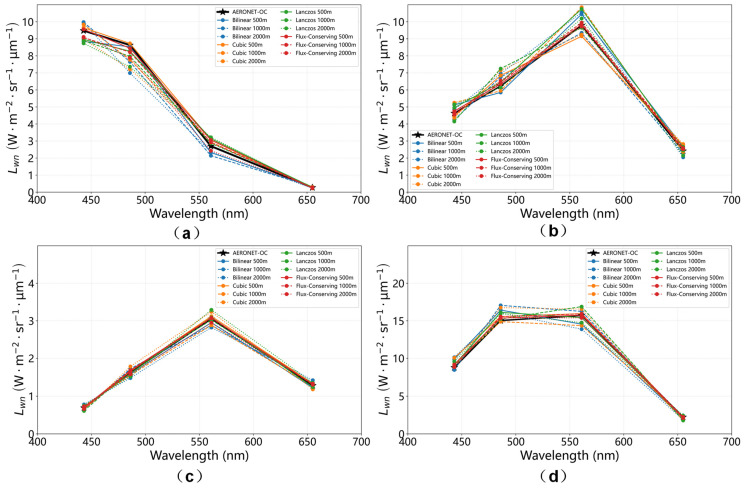
Multi-site comparison of normalized water-leaving radiance retrieved using various resampling methods based on Level 1.5 data. (**a**) GOT-Seaprism site; (**b**) MVCO site; (**c**) Helsinki_Lighthouse site; (**d**) Lucinda site.

Figure 14 and Table 5 present a comprehensive evaluation of resampling performance across four geographically distinct aquatic sites, revealing consistent patterns in radiometric behavior. Bilinear interpolation consistently produces higher relative errors, particularly in the red band (655 nm), and these errors increase with coarser spatial resolutions, indicating strong sensitivity to boundary effects in heterogeneous waters. Cubic convolution performs competitively in some cases but shows substantial variability across sites, reflecting its dependence on local radiometric structure. Lanczos resampling achieves moderate accuracy but demonstrates a systematic underestimation of reflectance across most bands.

In contrast, the flux-conserving (FC) method exhibits overall superior radiometric stability, achieving the lowest errors in 32 of the 48 evaluated band–resolution–site combinations. Notably, FC maintains relative errors within ±5% across the blue–green spectral range (443–561 nm) for the majority of sites and resolutions, indicating reliable radiometric preservation across spatial scales. Accuracy degrades slightly at the coarsest resolution (2000 m), as exemplified by the −10.0% error at 561 nm for GOT-Seaprism, reflecting the challenges of extreme pixel aggregation.

In addition, the optimal resampling method occasionally varies with site and spectral band. At the Lucinda site, characterized as clear, optically deep water, Lanczos achieved slightly lower relative errors in the blue band (443 nm) at 500 m resolution. A similar pattern is observed at the Helsinki Lighthouse site (500 m, 443–486 nm), where cubic convolution or Lanczos performs competitively.

Interpolation-based methods also occasionally outperform FC in select red-band cases (655 nm), such as GOT-Seaprism and Lucinda at 500 m resolution, as well as in some coarser-resolution combinations (1000–2000 m) for several sites. These occurrences likely reflect relatively homogeneous radiometric structures with weak spatial gradients, where interpolation-based smoothing can incidentally reduce high-frequency noise, residual atmospheric effects, or edge-induced errors near land–water boundaries.

Despite these minor variations, the differences are small (typically within ±2–3%) and do not alter the overall conclusion that FC provides robust radiometric stability across diverse aquatic regimes.

Finally, the persistent negative bias in the red band (655 nm) observed across multiple sites is likely due to residual errors in atmospheric correction rather than the resampling process itself, highlighting the need for targeted refinement in red-band atmospheric processing.

## 5. Discussion

### 5.1. Radiometric Calibration Uncertainty Analysis

In this study, radiometric calibration uncertainty is discussed in terms of three primary sources: sensor observation errors, uncertainties associated with the atmospheric correction process, and scale-related effects.

#### 5.1.1. Sensor Radiometric Calibration Uncertainty

The radiometric calibration of Landsat-8 OLI is subject to instrument-level uncertainties, including detector responsivity, electronic noise, optical degradation, and onboard calibration accuracy. The absolute radiometric uncertainty in the visible bands is reported to be approximately 2–3% [42], consistent with values commonly adopted in aquatic remote sensing. This uncertainty is primarily systematic, spatially uniform, and spectrally dependent. In the context of Rayleigh scattering calibration, it sets a baseline for absolute TOA reflectance uncertainty. Since all resampling experiments use the same calibrated Level-1 data, sensor-related errors remain constant across spatial resolutions and methods, mainly affecting the absolute reflectance values while having minimal impact on the relative evaluation of resampling performance.

#### 5.1.2. Atmospheric Correction Uncertainty

In the Rayleigh scattering calibration framework of this study, the uncertainty in the atmospheric correction process mainly arises from two sources: errors in AOD estimation and discretization and interpolation effects in the radiative transfer LUT. Under the marine atmospheric conditions considered here, these two factors are regarded as the dominant contributors to atmospheric correction uncertainty, while other atmospheric parameters are held constant. It should be noted that water vapor and ozone were treated as spatially uniform in this analysis; however, this assumption is reasonable for the selected scenes under marine atmospheric conditions, spatial variability in these constituents may introduce additional uncertainty when the method is applied over broader spatial domains. Assuming the two sources are independent, the total atmospheric correction uncertainty was estimated by combining their individual contributions as:

(17) $$\begin{array}{c} \delta_{ATM}=\sqrt{{\left(\delta_{AOD}\right)}^{2}+{\left(\delta_{LUT}\right)}^{2}}. \end{array}$$

1. AOD Uncertainty

The AOD uncertainty was quantified by introducing a fixed bias (ΔAOD = 0.01) to the AOD input in the 6S radiative transfer model. A maritime aerosol model was selected in accordance with the oceanic calibration environment. With all other atmospheric and geometric parameters held constant, the resulting variation in simulated TOA reflectance yielded the spectral AOD uncertainty for each band.

2. LUT Interpolation Uncertainty

The LUT uncertainty was quantified by comparing TOA reflectance obtained from LUT interpolation with that directly simulated by the 6S model under identical atmospheric and geometric conditions. All LUT input parameters were kept consistent between the two approaches, such that the observed differences primarily reflect LUT discretization and interpolation effects.

As shown in Table 6, the atmospheric correction uncertainty remains below 2% across all spectral bands. LUT interpolation generally contributes slightly more to the total uncertainty than AOD perturbations, particularly at shorter wavelengths (443–486 nm), reflecting the higher sensitivity of shortwave bands to discretization and interpolation effects. In longer wavelengths (561–655 nm), both sources contribute less, resulting in overall smaller uncertainties. These results indicate that the combined atmospheric correction uncertainty is relatively limited across the visible spectrum, supporting the reliability of TOA reflectance retrieval and subsequent water-leaving radiance estimation in the Rayleigh scattering calibration framework.

#### 5.1.3. Spatial Resolution Uncertainty

To evaluate uncertainty related to spatial resolution, the original 30 m images were resampled to coarser resolutions of 500, 1000, and 2000 m using the flux-conserving (FC) method. This approach preserves radiometric flux, so that differences in reflectance mainly result from pixel aggregation and spatial mixing.

For each spectral band, the resampled reflectance was compared with the original 30 m reflectance, which was treated as a reference representation of surface reflectance. The uncertainty was quantified using the relative difference:

(18) $$\begin{array}{c} \delta_{SR}\left(\lambda \right)=\frac{\left|\rho_{\lambda }^{resampled}-\rho_{\lambda }^{30m}\right|}{\rho_{\lambda }^{30m}}\times 100\%. \end{array}$$

The resulting values were spatially averaged over the study area for each spectral band and resolution. The spatially averaged uncertainties are summarized in Table 7.

Table 7 shows that uncertainty increases as spatial resolution decreases. Shorter wavelengths (443 and 486 nm) exhibit slightly higher uncertainties, whereas longer wavelengths remain more stable. This indicates that image scale is an important factor affecting radiometric measurements at kilometer-level resolutions.

### 5.2. Flux-Conserving Resampling in Diverse Aquatic Conditions

The superior performance of the flux-conserving (FC) resampling method in optically heterogeneous waters, as established in Section 4, positions it as a promising alternative to interpolation-based techniques. In aquatic remote sensing, however, the efficacy of any spatial processing method is inherently governed by the water body’s inherent optical properties, which vary dramatically across environments. To critically assess the FC method’s general applicability and to address the need for validation under such extreme or distinct optical regimes, we rigorously evaluated its performance across four strategically selected validation sites (GOT-Seaprism, MVCO, Helsinki Lighthouse, and Lucinda). Chosen to represent a broad spectrum of key aquatic optical conditions, these sites provide imagery of distinct, canonical regimes, enabling a targeted assessment of the FC method under different dominant optical processes.

The GOT-Seaprism site in the Gulf of Thailand represents highly turbid nearshore waters with pronounced spatial heterogeneity. The MVCO site along the northeastern coast of the United States corresponds to temperate coastal waters influenced by seasonal phytoplankton growth and variations in suspended particulate matter. The Helsinki Lighthouse site in the Baltic Sea characterizes waters dominated by colored dissolved organic matter (CDOM) absorption. Finally, the Lucinda site in Australian offshore waters represents relatively clear, optically deep waters with a low absorption background, serving as a reasonable proxy for oligotrophic open-ocean optical conditions.

Pixel-level comparisons of water-leaving radiance were performed at three spatial resolutions (500 m, 1000 m, and 2000 m). The RMSE and R^2^ were employed as evaluation metrics to quantify absolute radiometric deviation and the preservation of spatial patterns, respectively. The comprehensive results for each site and resolution are presented in Table 8.

Overall, FC consistently achieves low RMSE and high R^2^ values, even at the coarsest spatial resolution (2000 m). A slight performance degradation occurs as pixel size increases, which reflects the inherent challenge of subpixel heterogeneity. Despite this, the method maintains an R^2^ above 0.83 for all sites. Differences among sites correspond closely to their optical characteristics: high-turbidity nearshore waters (GOT-Seaprism) and CDOM-dominated waters (Helsinki Lighthouse) exhibit relatively higher RMSE, whereas the optically deep and clear offshore site (Lucinda) shows the lowest errors, highlighting the effectiveness of FC resampling under low-absorption, homogeneous conditions.

These results indicate that the FC method reliably preserves radiometric fidelity across diverse water types and spatial resolutions. Its flux-conserving principle mitigates nonlinear radiometric effects induced by pixel aggregation, making it particularly robust in heterogeneous or optically extreme waters. Future work should extend this validation to more dynamic conditions, such as sediment-laden river plumes or intense algal blooms, to further define the boundaries of the method’s robustness.

It is worth noting that the resampling analysis in this study focused on visible bands (443–655 nm), as these wavelengths are most directly relevant to Rayleigh scattering calibration and water-leaving radiance retrieval. Near-Infrared (NIR) and Short-Wave Infrared (SWIR) bands, however, play an equally critical role in atmospheric correction and turbid water detection. Extending this evaluation to include NIR and SWIR bands, therefore, represents an important next step toward assessing the full-spectrum performance of flux-conserving resampling.

Additionally, although scenes with cloud cover below 10% were selected, residual thin clouds may still introduce some uncertainty in radiometric consistency at coarse resolutions, particularly at 2000 m. Future studies could explicitly quantify this effect or adopt advanced atmospheric correction methods, such as neural-network-based approaches near clouds [43], to further improve validation under less ideal conditions.

### 5.3. Implications for Long-Term Ocean Color Time Series Stability

Building on the multi-scale validation results, the implications for long-term ocean color monitoring are considered here. In addition to its impact on instantaneous radiometric accuracy, the choice of spatial resampling method may also influence the stability of long-term ocean color time series. Many ocean color applications, including water quality assessment, climate-related trend detection, and interannual variability analysis, rely on consistent radiometric behavior across extended temporal records. When multi-year datasets involve varying spatial resolutions or multi-sensor integration, inconsistencies in spatial processing may introduce subtle systematic deviations that propagate into trend analyses.

As demonstrated in Section 4 and Section 5.2, the flux-conserving (FC) method maintains radiometric fidelity across different spatial scales and optical environments. Because it preserves the integrated radiometric flux during spatial aggregation, changes in pixel size primarily reflect spatial mixing effects rather than artificial redistribution of radiance. This property is particularly relevant in long-term monitoring scenarios where spatial resolution may vary between datasets or processing stages.

Interpolation-based methods, while computationally efficient, inherently perform weighted averaging of neighboring pixels. In optically heterogeneous coastal waters, such operations may alter the radiometric balance in a scale-dependent manner. If applied inconsistently across different segments of a temporal record, these effects may introduce artificial discontinuities or scale-dependent variability that is not directly related to environmental change.

In contrast, the FC approach ensures that aggregated reflectance values remain physically consistent representations of the original radiometric signal. Although it does not eliminate subpixel heterogeneity, it minimizes additional methodological uncertainty associated with spatial transformation. This characteristic may contribute to improved comparability across multi-resolution datasets and reduce the risk of bias in long-term trend estimation, thereby enhancing the reliability of climate-quality ocean color data records.

The present study focuses on instantaneous radiometric comparisons. A dedicated multi-temporal evaluation, incorporating extended time series and statistical trend metrics, would be valuable for quantifying the cumulative influence of spatial resampling on long-term ocean color variability. Such analysis would further clarify the role of spatial preprocessing in the interpretation of decadal-scale bio-optical changes and help establish best practices for consistent time series generation.

## 6. Conclusions

Conventional studies on multi-source aquatic remote sensing have primarily focused on image fusion strategies or bio-optical inversion algorithms, whereas the spatial resampling step preceding fusion has generally been treated as a technical detail. In practice, however, resampling is unavoidable and always occurs before any fusion or retrieval procedure. This study shifts the analytical focus to this often-overlooked preprocessing stage and systematically evaluates how different resampling strategies influence radiometric consistency in water color remote sensing. The main contribution lies in revealing the distortions induced by resampling across spatial scales and in demonstrating the advantages of the flux-conserving method to mitigate these distortions.

The principle of energy conservation is particularly relevant for ocean color remote sensing because aquatic signals are inherently weak and highly sensitive. Water bodies are low-reflectance targets, and radiometric information in the blue and green bands, which is essential for retrieving bio-optical parameters, can be significantly affected even by minor perturbations. Maintaining energy conservation during spatial resampling helps suppress the amplification of such small errors and preserves the fidelity of weak aquatic signals, ensuring more reliable retrieval of water quality indicators.

Quantitative evaluation using Landsat-8 OLI imagery confirms that the FC method consistently outperforms conventional interpolation techniques across all spatial resolutions and spectral bands. Under the most challenging condition examined, namely downsampling from 30 m to 2000 m in the blue band (443 nm), the FC method reduced the mean relative radiometric error to 4.36%, compared with 7.80% for bilinear interpolation, representing an error reduction of approximately 44%. The corresponding standard deviation decreased from 5.31% to 3.59%, indicating substantial improvement in radiometric stability under severe mixed-pixel conditions. Similar advantages were observed in water-leaving radiance retrieval across four geographically diverse AERONET-OC sites, where FC maintained relative errors within ±5% across the blue–green bands for the majority of site and band combinations, and coefficients of determination remained above 0.83, demonstrating robust preservation of spatial patterns and radiometric fidelity even at coarse resolutions.

Multi-scale and multi-regional validation further indicates that the performance of the FC method is largely insensitive to variations in bio-optical conditions. Consistent results were obtained across environments ranging from tropical clear waters to high-latitude turbid systems, highlighting its strong generalization capability and robustness with respect to variations in water constituents and aquatic remote sensing products. The proposed framework also offers a valuable reference for the vicarious calibration of low-resolution sensors and for cross-calibration between sensors with differing spatial resolutions.

Several limitations should be acknowledged when interpreting these results. First, the present analysis is based on Landsat-8 OLI imagery, a sensor characterized by high radiometric stability and a favorable signal-to-noise ratio. Accordingly, the relative performance of the flux-conserving method compared with interpolation-based approaches may vary when applied to sensors with different radiometric characteristics and noise levels. For example, Sentinel-2 MSI, MODIS, and VIIRS differ in signal-to-noise ratio, spatial resolution, and spectral response functions, which may influence the relative magnitude of resampling-induced uncertainty.

Second, although the four AERONET-OC validation sites encompass a broad range of bio-optical conditions—from tropical clear waters to high-latitude turbid systems—they do not fully represent the most optically extreme aquatic environments encountered globally. River-influenced coastal margins with sharp turbidity gradients, glacial meltwater plumes, and nearshore zones with pronounced sub-pixel variability are examples of highly heterogeneous settings in which resampling-induced errors may be amplified.

In addition, the atmospheric correction in this study assumed spatially uniform water vapor and ozone concentrations. While this assumption is reasonable for the selected marine scenes, it may introduce additional uncertainty when the method is applied over broader spatial domains where these constituents exhibit significant spatial variability.

Finally, this study deliberately isolated the effects of spatial resampling under controlled observational geometries. Future work should examine how flux-conserving resampling interacts with viewing geometry effects, including the bidirectional reflectance distribution, and extend validation to more complex atmospheric conditions, such as seasonal cycles, and to additional satellite platforms. Such efforts would provide a more comprehensive assessment of the method’s operational applicability for global aquatic remote sensing.

## Figures and Tables

**Table 1:** Spectral characteristics of Landsat-8 OLI bands ^1^.

Band Number	Band Name	Central Wavelength (nm)	Spatial Resolution (m)
1	Coastal/Aerosol	443	30
2	Blue	486	30
3	Green	561	30
4	Red	655	30
5	Near-infrared (NIR)	865	30
6	Shortwave infrared 1 (SWIR-1)	1609	30
7	Shortwave infrared 2 (SWIR-2)	2201	30
8	Panchromatic	592	15
9	Cirrus	1373	30

^1^ From USGS Landsat 8 Data Users Handbook, Version 5.0 (2019).

**Table 2:** Landsat-8 OLI scenes matched with AERONET-OC stations.

Acquisition Date (UTC)	Path/Row	Scene Center Coordinates	Cloud Cover (%)	Matched Station
15 December 2015	128/54	(8.67° N, 101.05° E)	6.83	GOT–Seaprism
23 April 2018	11/31	(41.75° N, 69.81° W)	3.00	MVCO
28 June 2019	189/18	(60.07° N, 23.09° E)	3.50	Helsinki_Lighthouse
8 February 2021	95/73	(18.79° S, 146.10° E)	6.28	Lucinda

**Table 3:** Input parameters for the LUT simulations.

Parameter (Unit)		Values	Interval
Central Wavelength (nm)		443 nm, 486 nm, 561 nm, 655 nm	Fixed
Solar Zenith Angle (°)		0~80°	5°
Viewing Zenith Angle (°)		0~7.5°	1.5°
Relative Azimuth Angle (°)		0~180°	10°
Aerosol Optical Depth		1 × 10^−6^~0.5 at 550 nm	0.03 (AOD < 0.3) 0.05 (AOD ≥ 0.3)
Aerosol Model		Maritime	-
Wind Speed (m s^−1^)		0~5	0.5
Atmospheric Profile	Water Vapor Content (g cm^−2^)	3.75	Fixed
	Ozone Concentration (DU)	547.46 DU	Fixed

**Table 4:** Statistical comparison of relative errors (%) between observed and calibrated TOA radiance across different bands and spatial resolutions.

Band	Resolution	Average Relative Error/%				STD of Relative Error/%			
		BL	CC	Lanczos	FC	BL	CC	Lanczos	FC
443 nm	500 m	5.90	6.74	7.30	5.71	3.79	3.95	4.07	3.77
	1000 m	7.37	8.12	7.52	5.70	5.07	5.06	4.92	4.36
	2000 m	7.80	7.17	6.93	4.36	5.31	5.20	4.86	3.59
486 nm	500 m	5.26	6.03	5.60	4.37	3.81	4.45	4.12	3.48
	1000 m	6.37	6.26	6.24	5.57	5.26	5.50	5.56	4.77
	2000 m	6.08	7.31	6.81	5.24	5.62	6.36	5.78	4.91
561 nm	500 m	4.12	4.56	4.15	4.17	3.75	4.38	3.86	3.91
	1000 m	5.07	5.74	5.08	4.16	5.25	5.79	4.60	4.45
	2000 m	5.51	5.58	5.75	4.78	5.25	5.66	5.76	4.64
655 nm	500 m	4.38	4.57	4.59	4.33	4.01	4.44	4.10	3.84
	1000 m	5.36	5.14	4.22	5.18	5.15	5.38	4.98	5.00
	2000 m	5.37	5.34	5.76	4.81	5.47	5.72	5.83	4.38

Note: BL, bilinear interpolation; CC, cubic convolution; FC, flux-conserving resampling.

**Table 5:** Optimal resampling method and relative error (%) of TOA radiance by site, spectral band, and spatial resolution.

Site	Resolution	Band			
		443 nm	486 nm	561 nm	655 nm
GOT-Seaprism	500 m	FC (0.8%)	CC (1.2%)	FC (13.3%)	Lanczos (5.9%)
	1000 m	CC (3.8%)	FC (−4.8%)	Lanczos (6.3%)	Lanczos (8.1%)
	2000 m	FC (−3.9%)	FC (−8.6%)	FC (−10.0%)	FC (6.7%)
MVCO	500 m	FC (2.6%)	FC (1.9%)	FC (0.6%)	Lanczos (1.2%)
	1000 m	FC (−1.7%)	FC (3.5%)	Lanczos (−1.4%)	CC (−2.9%)
	2000 m	FC (0.4%)	Lanczos (−1.3%)	FC (−0.7%)	FC (6.6%)
Helsinki_Lighthouse	500 m	CC (−1.4%)	Lanczos (−1.2%)	FC (0.7%)	FC (2.3%)
	1000 m	FC (−4.3%)	FC (1.9%)	Lanczos (−1.0%)	FC (1.6%)
	2000 m	FC (5.8%)	FC (−2.5%)	FC (−3.3%)	FC (3.9%)
Lucinda	500 m	Lanczos (3.8%)	FC (2.1%)	FC (0.3%)	Lanczos (−2.2%)
	1000 m	FC (0.7%)	CC (−1.0%)	FC (1.4%)	FC (0.9%)
	2000 m	CC (1.5%)	FC (2.4%)	FC (−2.0%)	FC (−5.8%)

Note: The table lists the optimal resampling method and its relative error for each combination of spectral band, spatial resolution, and validation site. Positive values indicate overestimation, negative values indicate underestimation. FC: Flux-Conserving; CC: Cubic Convolution.

**Table 6:** Atmospheric correction uncertainty contributions and combined uncertainty of TOA reflectance for different spectral bands.

Band (nm)	$\delta_{\;AOD\;}$(%)	$\delta_{\;LUT\;}$(%)	$\delta_{\;ATM\;}$(%)
443	1.08	1.66	1.98
486	1.05	1.39	1.74
561	0.83	1.01	1.31
655	0.67	0.93	1.15

**Table 7:** Spatial resolution uncertainty of TOA reflectance for different spectral bands at 500 m, 1000 m, and 2000 m resolutions.

Band (nm)	$\delta_{\;SR\;500m\;}$(%)	$\delta_{\;SR\;1000m\;}$(%)	$\delta_{\;SR\;2000m\;}$(%)
443	1.82	3.05	5.12
486	1.57	2.91	4.69
561	1.41	2.36	3.94
655	1.09	2.08	3.32

**Table 8:** Pixel-Level Water-Leaving Radiance Metrics for Flux-Conserving Resampling at Four Sites.

Site	Resolution (m)	RMSE	R^2^
GOT-Seaprism	500 m	0.006	0.920
	1000 m	0.009	0.883
	2000 m	0.011	0.845
MVCO	500 m	0.005	0.930
	1000 m	0.006	0.897
	2000 m	0.009	0.862
Helsinki_Lighthouse	500 m	0.007	0.904
	1000 m	0.009	0.860
	2000 m	0.013	0.833
Lucinda	500 m	0.004	0.952
	1000 m	0.005	0.926
	2000 m	0.008	0.889

## Data Availability

The datasets analyzed in this study are publicly available from NASA AERONET-OC (https://aeronet.gsfc.nasa.gov) (accessed on 26 February 2026), NASA GES DISC (https://disc.gsfc.nasa.gov) (accessed on 26 February 2026), and ECMWF (https://www.ecmwf.int) (accessed on 26 February 2026).

## Abbreviations

The following abbreviations are used in this manuscript:

FC	Flux-Conserving (resampling method)
BL	Bilinear interpolation
CC	Cubic convolution interpolation
AOD	Aerosol Optical Depth
TOA	Top of Atmosphere
6S	Second Simulation of a Satellite Signal in the Solar Spectrum
LUT	Lookup Table
BRDF	Bidirectional Reflectance Distribution Function
RMSE	Root Mean Square Error
R^2^	Coefficient of Determination

## References

[1] McCarthy M.J., Herrero H.V., Insalaco S.A., Radabaugh K.R., Moyer R.P., Muller-Karger F.E. (2025). Satellite remote sensing for environmental sustainable development goals: A review of applications for terrestrial and marine protected areas. Remote Sens. Appl. Soc. Environ..

[2] Zhao S., Liu M., Tao M., Zhou W., Lu X., Xiong Y., Li F., Wang Q. (2023). The role of satellite remote sensing in mitigating and adapting to global climate change. Sci. Total Environ..

[3] Adjovu G.E., Stephen H., James D., Ahmad S. (2023). Overview of the Application of Remote Sensing in Effective Monitoring of Water Quality Parameters. Remote Sens..

[4] Kaplan G., Yalcinkaya F., Altıok E., Avdan Z.Y., Avdan U. (2024). The role of remote sensing in the evolution of water pollution detection and monitoring: A comprehensive review. Phys. Chem. Earth.

[5] Yang Y., Wang Z., Chen P., Shen X., Kong W., Huang G., Shu R. (2024). High-Resolution Ocean Color Reconstruction and Analysis Focusing on Kd490 via Machine Learning Model Integration of MODIS and Sentinel-2 (MSI). Front. Mar. Sci..

[6] Yang H., Kong J., Hu H., Du Y., Gao M., Chen F. (2022). A Review of Remote Sensing for Water Quality Retrieval: Progress and Challenges. Remote Sens..

[7] Loveland T.R., Irons J.R. (2016). Landsat 8: The plans, the reality, and the legacy. Remote Sens. Environ..

[8] Tahersima M.H., Thome K., Wenny B.N., Voskanian N., Yarahmadi M. (2023). Intercomparison of Landsat OLI and JPSS VIIRS Using a Combination of RadCalNet Sites as a Common Reference. Remote Sens..

[9] Campbell J.B., Wynne R.H. (2011). Introduction to Remote Sensing.

[10] Samadzadegan F., Toosi A., Javan F.D. (2024). A Critical Review on Multi-Sensor and Multi-Platform Remote Sensing Data Fusion Approaches: Current Status and Prospects. Int. J. Remote Sens..

[11] Xiao J., Aggarwal A.K., Duc N.H., Arya A., Rage U.K., Avtar R. (2023). A review of remote sensing image spatiotemporal fusion: Challenges, applications and recent trends. Remote Sens. Appl. Soc. Environ..

[12] Zhang G., Liang S., Ma H., Jiang B., Wang J., Zhao X., He T. (2024). Simultaneous estimation of five temporally regular land variables at seven spatial resolutions from seven satellite data using a multi-scale and multi-depth convolutional neural network. Remote Sens. Environ..

[13] Wang S., Zhou Q. (2024). Multi-source fusion enhanced feature segmentation in remote sensing imagery. ISPRS Ann. Photogramm. Remote Sens. Spat. Inf. Sci..

[14] Zhang J. (2010). Multi-Source Remote Sensing Data Fusion: Status and Trends. Int. J. Image Data Fusion.

[15] Zhang Z.W., Yu T., Meng Q.Y., Hu X., Li C. (2013). Study on the Impact of Spatial Resampling Methods on Remote Sensing Image Information. J. Cent. China Norm. Univ. Nat. Sci..

[16] Yang X.Y., Zhao T. (2012). Analysis of Water Extraction Effect from Remote Sensing Images at Different Scales. Groundwater.

[17] Chen J., Chen Z., Huang R., You H., Han X., Yue T., Zhou G. (2023). The Effects of Spatial Resolution and Resampling on the Classification Accuracy of Wetland Vegetation Species and Ground Objects: A Study Based on High Spatial Resolution UAV Images. Drones.

[18] Roy D.P., Li J., Zhang H.K., Yan L. (2016). Best Practices for the Reprojection and Resampling of Sentinel-2 Multi Spectral Instrument Level 1C Data. Remote Sens. Lett..

[19] Porwal S., Katiyar S.K. (2014). Performance Evaluation of Various Resampling Techniques on IRS Imagery. Proceedings of the 2014 Seventh International Conference on Contemporary Computing (IC3), Noida, India, 7–9 August 2014.

[20] Tian Y., Wang Z. (2025). A Spectral-Preserving Resampling for Spatial Upscaling of Hyperspectral Imagery. Sci. Remote Sens..

[21] Sales V., Marques A., Racolte G., Schimalski M.B., Liesenberg V. (2023). Evaluation of resampling techniques to provide better synthesized input data to Super-Resolution deep learning model training. Proceedings of the IGARSS 2023–2023 IEEE International Geoscience and Remote Sensing Symposium, Pasadena, CA, USA, 16–21 July 2023.

[22] Huang M., Xing X., Luo W., Wang Z. (2019). Recalibration of Offshore Chlorophyll Content Based on Virtual Satellite Constellation. Proceedings of the IGARSS 2019—2019 IEEE International Geoscience and Remote Sensing Symposium, Yokohama, Japan, 28 July–2 August 2019.

[23] Kim H.C., Lee S.-M., Chai T., Ngan F., Pan L., Lee P. (2018). A Conservative Downscaling of Satellite-Detected Chemical Compositions: NO_2_ Column Densities of OMI, GOME-2, and CMAQ. Remote Sens..

[24] Anutaliya A. (2023). Surface Circulation in the Gulf of Thailand from Remotely Sensed Observations: Seasonal and Interannual Timescales. Ocean Sci..

[25] Roy D.P., Wulder M.A., Loveland T.R., Woodcock C.E., Allen R.G., Anderson M.C., Helder D., Irons J.R., Johnson D.M., Kennedy R. (2014). Landsat-8: Science and Product Vision for Terrestrial Global Change Research. Remote Sens. Environ..

[26] U.S. Geological Survey (2019). Landsat 8 (L8) Data Users Handbook, Version 5.0.

[27] Zibordi G., Mélin F., Berthon J.F., Holben B., Slutsker I., Giles D., D’Alimonte D., Vandemark D., Feng H., Schuster G. (2009). AERONET-OC: A Network for the Validation of Ocean Color Primary Products. J. Atmos. Ocean. Technol..

[28] Irons J.R., Dwyer J.L., Barsi J.A. (2012). The Next Landsat Satellite: The Landsat Data Continuity Mission. Remote Sens. Environ..

[29] Koepke P. (1984). Effective Reflectance of Oceanic Whitecaps. Appl. Opt..

[30] Sutherland I.E., Hodgman G.W. (1974). Reentrant Polygon Clipping. Commun. ACM.

[31] McGlynn T.A. (2002). A Fast Flux-Conserving Resampling Algorithm. American Astronomical Society Meeting Abstracts.

[32] Song W., Hu Q., Liu S., Hou Y. (2025). Decadal Spatiotemporal Dynamics of Surface Water Bodies in Zhengzhou, China: Remote Sensing Monitoring and Analysis of Driving Factors. Sci. Rep..

[33] Zhu Z., Wang S., Woodcock C.E. (2015). Improvement and Expansion of the Fmask Algorithm: Cloud, Cloud Shadow, and Snow Detection for Landsats 4–7, 8, and Sentinel 2 Images. Remote Sens. Environ..

[34] Kay S., Hedley J.D., Lavender S. (2009). Sun Glint Correction of High and Low Spatial Resolution Images of Aquatic Scenes: A Review of Methods for Visible and Near-Infrared Wavelengths. Remote Sens..

[35] Mondejar J.P., Tongco A.F. (2019). Near Infrared Band of Landsat 8 as Water Index: A Case Study around Cordova and Lapu-Lapu City, Cebu, Philippines. Sustain. Environ. Res..

[36] Zhou L.G., Ma W.C., Gu W.H., Huai H.Y. (2011). Atmospheric Correction of HJ-1 CCD Data for Water Imagery Based on Dark Object Model. Spectrosc. Spect. Anal..

[37] Zhao W., Chen G.M., Niu S.L. (2013). Rayleigh Scattering Calibration Research of Chinese Ocean Color Sensor. Acta Oceanol. Sin..

[38] Chen F.N., Fan Y.Z., Hong J., Huang C., Li S., Yang B.Y., Tu B.H., Han L., Sun B. (2020). In-Flight Radiation Calibration of a Directional Polarimetric Camera at Visible Bands Onboard GF-5. Acta Opt. Sin..

[39] Pan Y.W., Chen J.J., Sun L., Zhang P.F., Chen F.N., Meng B.H., Xiang G.F., Hong J. (2024). Ocean Sites Calibration and Water Color Retrieval for Directional Polarimetric Camera. Acta Opt. Sin..

[40] Wang D., Feng X., Ma R., Kang G. (2007). A Method for Retrieving Water-Leaving Radiance from Landsat TM Image in Taihu Lake, East China. Chin. Geogr. Sci..

[41] Tian L., Zhang T.L., Chen S.G., Shu C. (2014). Comparison of Flux-Conserving Resampling Method with Commonly Used Methods in Merging of Remote Sensing Data. Period. Ocean Univ. China.

[42] U.S. Geological Survey Landsat Radiometric Uncertainty. Landsat Missions. https://www.usgs.gov/landsat-missions/landsat-radiometric-uncertainty.

[43] Men J., Li W., Chen X., Feng L., Tian L. (2026). Improving ocean color atmospheric correction near clouds with neural network. IEEE Trans. Geosci. Remote Sens..

